# Phloretin-nanospanlastics for targeting the Akt/PI3K signaling pathways in dimethylhydrazine-induced colon cancer in mice

**DOI:** 10.1016/j.ijpx.2024.100311

**Published:** 2024-12-17

**Authors:** Ebtsam A. Abdel-Wahab, Zahraa Haleem Al-Qaim, Ahmed T.H. Faris Al-Karkhi, Aysam M. Fayed, Ahmed M. Eldmrdash, Mohammed Abdalla Hussein, Amal Abdel-Aziz, Azza M. Metwaly, Heba.G. Abdelzaher, M.A. Abdelzaher, Diana A. ALsherif

**Affiliations:** a Department of Biophysics, Faculty of Applied Health Sciences, October 6 University, Egypt; b Anesthesia Techniques Department, College of Health and Medical Techniques, Al-Mustaqbal University, 51001 Babylon, Iraq; c College of Medicine, Al-Mustaqbal University, 51001 Babylon, Iraq; d Medical Laboratories Techniques Department, AL-Mustaqbal University, 51001 Hillah, Babil, Iraq; e Molecular Biology Department, Genetic Engineering and Biotechnology Research Institute, University of Sadat City, Sadat City, Egypt; f Department of Medical Labs, Faculty of Applied Medical Sciences Technology, October 6 University, Egypt; g Department of Biotechnology, Faculty of Applied Health Sciences Technology, October 6 University, Egypt; h Department of Clinical pharmacy, Faculty of Pharmacy, Minia University, 61519 Minia, Egypt; i Environmental Science and Industrial Development Department, Faculty of Postgraduate Studies for Advanced Sciences, Beni-Suef University, Beni-Suef 62511, Egypt; j Technology of Radiology and Medical Imaging Department, Faculty of Applied Health Science Technology, October 6 University, Egypt

**Keywords:** Colon Cancer, Phloretin-Nanospanlastics, Akt/PI3K, Dimethylhydrazine, Inflammatory Mediators, Antioxidants

## Abstract

**Objectives:**

Colorectal cancer is the third most common cancer worldwide, accounting for approximately 10 % of all cancer cases. It is also the second leading cause of cancer-related deaths globally. Phloretin is a natural compound found in apples and other fruits. It has been studied for its potential health benefits, including antioxidant and anti-inflammatory properties. However, more research is needed to fully understand its impact on cancer prevention or treatment. This article aimed to prepare phloretin-nanospanlastics (Ph-NSLs) to evaluate their effects on dimethylhydrazine (DMH)-induced colon cancer in mice.

**Methods:**

Morphology, Particle size, zeta potential, UV–vis, entrapment efficiency, polydispersity index, FT-IR spectra, and drug release of phloretin and Ph-NSLs at pH 6.8.were described. Ph-NSLs were also tested for their anti-cancer properties in DMH-induced colon cancer in mice. A 36 mice were divided into 6 groups; Normal control, DMH (20 mg/k.g.b.w.), DMH + Ph-NSLs (25 mg/k.g.b.w.), DMH + Ph-NSLs (50 mg/k.g.b.w.), DMH + 5-FU(20 mg/k.g.b.w.), DMH + Ph-NSLs (50 mg), 5-FU (20 mg). Ph-NSLs were tested for their anticancer properties in DMH-treated mice by evaluating the IC50, viability and inhibitory values of Ph-NSLs against Caco-2. Also, the effect of Ph-NSLs administration on number of surviving mice, number of tumors/mice, average of tumor size, Hb, RBCs, WBCs, C19–9, MDA, GSH, SOD, IL-2, TNF-α, TGF-β1, CEA, and P53 levels in mice treated DMH were estimated.

**Results:**

The synthesized Ph-NSLs were uniform, spherically shaped, and well dispersed, with a size, entrapment efficiency, and polydispersity index of approximately 114.06 ± 8.35 nm, 78.60 %, and 0.05, respectively. The zeta potential value of Ph-NSLs was measured at −21.5 ± 1.47 mV. Zeta potential reflects the surface charge of nanoparticles and affects their stability and interactions. UV spectra of phloretin and Ph-NSLs showed strong absorption peaks at 225 and 285 nm. These peaks correspond to specific wavelengths where the compound absorbs light. The percentage of Ph- NSLs release was found to be 56.87 ± 2.45 %. IC50 of Ph-NSLs was recorded 15.76 ± 0.42 μg/ml and the viability and inhibitory values of Ph-NSLs against Caco-2 cell lines was resorded 2.39, and 97.61 %, respectively at 100 μg/ml as well as 10.3, and 89.7 %, respectively at 50 μg/ml.

Moreover, The combination of 5-FU and Ph-NSLs resulted in a moderate increase in survival and significantly reduces tumor size and number, showing enhanced anticancer efficacy compared to individual treatments as well as attenuated levels of hemoglobin (Hb), red blood cells (RBCs), and white blood cells (WBCs). Reduced plasma cancer antigen 19–9 (CA19–9) levels as well as improved of colon malondialdehyde (MDA), reduced glutathione (GSH), superoxide dismutase (SOD), interleukine-2 (IL-2), tumor necrosis factor-alpha (TNF-α), tumor growth factor-beta1 (TGF-β1), carcinoembryonic antigen (CEA), and tumor protein (P53) levels. Also, Ph-NSLs and 5FU, either alone or together, decreased the expression of the Akt and PI3K genes in the colon. The combination of Ph-NSLs and 5FU showed more pronounced anticancer activity than Ph-NSLs administered individually.

**Conclusion:**

The combination of 5-FU and Ph-NSLs significantly enhances anticancer efficacy, reducing both the number of tumors and average tumor size more effectively than either treatment alone. This synergistic effect leverages 5-FU's inhibition of DNA synthesis and phloretin's induction of apoptosis and inhibition of cell proliferation, offering a promising approach for improved cancer treatment outcomes.

## 1 Introduction

Globally, colorectal cancer is the third most common and second most deadly (WHO, 2024). About 153,020 people were diagnosed with CRC in 2023, and 52,550 died. Under-50s will have 19,550 cases and 3750 fatalities (Siegel et al., 2023). Furthermore, it is projected that the global occurrence of CRC will increase by over twofold by 2035, with a particularly significant rise in less developed countries, mostly driven by a high increase in cases among the elderly population (Papamichael et al., 2015). The abnormal growth of stomach tissues is what causes colorectal cancer (CRC), a condition that specifically affects the colon or rectum. Furthermore, individuals who have chronic ulcerative colitis and Crohn's disease have a heightened susceptibility to acquiring colorectal cancer (Triantafillidis et al., 2009; Edwards et al., 2010).

Chemotherapy targets actively dividing malignant cells, which proliferate faster than healthy cells (Navarro et al., 2017; Edgar et al., 2007). Its application can lead to various side effects such as lack of appetite, hair loss, and blood abnormalities (Holaday and Berkowitz, 2009). Accordingly, based on WHO reports, around 80 % of the global population uses traditional medicines (Kooti and Daraei, 2017). Phytotherapy, often known as phytomedicine, is a medical method that utilizes certain plants or a mixture of plants to heal diseases. Medicinal plants can improve an individual's physical, psychological, and emotional well-being by reinstating the body's ability to protect, regulate, and restore itself (Marzouni et al., 2016; Sharma and Jain, 2009; Kalinowska et al., 2020).

Phloretin is a naturally occurring dihydrochalcone that can be found in common fruits, such as apples and strawberries. Prior studies have demonstrated that phloretin exhibits anti-inflammatory, antioxidant, and anti-cancer properties (Mariadoss et al., 2019a; Mariadoss et al., 2019b; Alsanea et al., 2017). Phloretin demonstrates anti-obesity effects by enhancing glucose regulation and insulin responsiveness and reducing the buildup of lipids in the liver (Wang et al., 2020). Despite this, phloretin's limited bioavailability and inability to dissolve properly in water limit its use in treating a wide range of illnesses. But some new papers suggest that we could make it easier for living things to absorb by making different dosage forms, like liposomes (Abu-Azzam and Nasr, 2020) and microemulsions (Fahmy et al., 2019).

Nanospanlastics (NSLs) are made up of two layers of surfactant molecules that surround a solution of an aqueous solute (Abdelrahman et al., 2017). Additionally, using a combination of edge activators (EAs), it is possible to create sorbitan-tailored vehicles with flexible walls (El Gizawy et al., 2019). EAs enhance elasticity by destabilizing and fluidizing the vesicular bilayers, thereby reducing interfacial tension. Therefore, these groundbreaking nano-cargo particles have the potential to accelerate the absorption of oral medications. The previous account discussed the utilization of NSLs to enhance drug distribution through the skin, eyes, duodenum, nails, and eardrums.

Additionally, the lack of papers on the subject indicates that there hasn't been much research on how Ph-NSLs can combat colon cancer. This is because dimethylhydrazine causes colon cancer in mice. As a continuation of our research program in the optimization of a new formula for medicinal applications (Abdelrahman et al., 2017; El Gizawy et al., 2019; El Gizawy and Hussein, 2017; Hussein, 2011; Hussein et al., 2021; Shehata et al., 2015; Hussein and Soad Mohamed, 2010; Mohamad et al., 2022), the objective of our work is to optimize the effectiveness of Ph-NSLs as well as enhance their oral bioavailability for DMH-induced mice colon carcinoma.

## 2 Materials and methods

### 2.1 Material

From Sigma-Aldrich, USA, Phloretin (99 %), DMH (97 %), span 80, tween 80, ethanol, chloroform, potassium chloride, sodium chloride, potassium dihydrogen phosphate, N-(e-dimethyl aminopropyl)-N-ethyl carbodiimide hydrochloride, dimethyl sulfoxide, and PBS were bought. Sodium tripolyphosphate, sodium hydroxide, and acetic acid were purchased from Bratachem, Indonesia.

### 2.2 Methods

#### 2.2.1 Preparation of Ph-NPs

Utilizing the thin-film hydration technique, we were able to successfully prepare NSLs that included phloretin. Using this method, a thin film was produced by adding 160 mg of span/tween 80 and 10 mg of phloretin to a mixture of chloroform and ethanol at a ratio of 1:1. Following this, a rotary evaporator was employed at a speed of 100 rpm (Kakkar and Kaur, 2011). The thin film was then hydrated with 10 ml of PBS with a pH of 7.4 that contained 1 ml of tween 80 to improve the dissolving of phloretin that was not encapsulated. After maturing the Ph-NPs overnight at 4 degrees Celsius, we employed bath sonication for five minutes to minimize the particle size of the Ph-NPs that were created for transmission electron microscopy (TEM) analysis (Fazil et al., 2012). After dropping a drop of colloidal solution on a 400 mesh-coated copper grid and letting the solvent evaporate in room-temperature air, we generated Ph-NSLss for transmission electron microscopy (TEM) measurements at both HRTEM and JEOL (JEM-2100 TEM).

#### 2.2.2 Zeta potential and particle size estimation

The determination of the average size, polydispersity index, and zeta potential of Ph-NPs took place using a dynamic laser scattering technique employing a Malvern Zeta sizer (Malvern Instruments, UK). Before measurements, water dispersion (950 uL) of nanoparticles (50 uL) took place to obtain 1 mL full volume, and results were recorded at 25 °C (Salar and Kumar, 2016).

#### 2.2.3 UV/Vis Spectroscopy

Using a spectrophotometer manufactured by Jasco in Japan, the UV–Vis absorption spectra of Ph-NPs and free phloretin were measured at a temperature of 25 degrees Celsius in the range of 200–400 nm at intervals of 1.0 nm. We performed the calculations on each record three times (Dickinson, 2012).

#### 2.2.4 Entrapment efficiency

The phloretin was entrapped using an ultracentrifuge (Branson Ultrasonics Corporation, CT, USA) by ultracentrifugation at 14,000 rpm for 45 min at 4 °C. We removed the phloretin-containing supernatant without touching the sample's sediment. The obtained supernatant was dissolved in a mixture of chloroform and methanol (40:60) using a vortex mixer. After mixing, we added an equal amount of the diluent to further dilute the sample. We measured the amount of unbound phloretin at 285 nm using a UV-VIS spectrophotometer. We conducted record calculations in triplicate (*n* = 3).

We calculated the phloretin entrapment efficiency (EE%) using the following equation:

$$\mathrm{EE}\;\left(\%\right)=\left[\left(\text{Total amount of phloretin}-\text{Free amount of phloretin}\right)/\text{Total amount of phloretin}\right]\times 100$$

#### 2.2.5 FT-IR spectroscopy

Measurements of FTIR spectra utilized using Bruker, Karlsruhe, Germany (Zhai et al., 2011). The spectra were captured with a resolution of 4 cm^−1^ at 400 to 4000 cm^−1^ at 25 °C.

#### 2.2.6 In vitro Ph-NPs release study

In vitro technique, the release of Ph-NPs was performed using the Tamilselvan and Raghavan methodology (Tamilselvan and Raghavan, 2014). In short, 10 mg of phloretin was mixed with 10 ml of phosphate buffer (pH 6.8) in a 50-mLvolumetric flask. The flask was then heated to 37 °C and shaken at 100 rpm in a biological shaker (LBS-030SLab Tech, Korea). We removed 0.5 ml aliquots at intervals of 0, 5, 10, 20, and 24 h, transferred them to a 1.5 ml microtube, and centrifuged them at 15,000 rpm (2015 Centurion Scientific, UK) for 10 min. We took the supernatant and analyzed it at 373 nm using UV–vis spectroscopy after a suitable dilution (Liu et al., 2016). We ran three duplicates of each experiment.

### 2.3 Biological study

#### 2.3.1 Mice

**Fig. 1:**
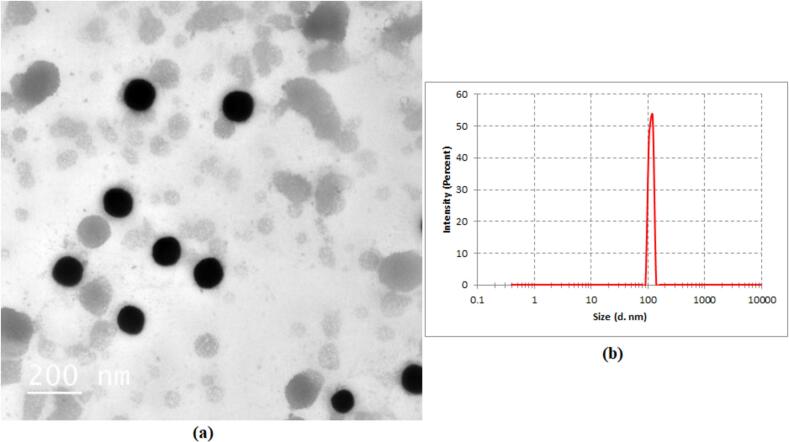
a and b: TEM image of Ph-NPs with scale bar 200 nm.

The instructions for this experiment were established by the Animal Care and Use Committee of the Faculty of Applied Health Science Technology at October 6 University in Egypt. A total of 48 mature mice, with an average weight of 35 ± 2.5 g, were acquired from the National Cancer Institute at Cairo University. The subjects were housed separately in cages within a climate-controlled room maintained at a temperature of 22 ± 2 °C, a relative humidity of 60 %, and an 8:00 to 20:00 light cycle. During the acclimation period, we provided each animal with a standard food ad libitum. The grouping plan is illustrated in Table 1. (See Fig. 1.)

**Table 1:** Grouping and treatment description.

No.	Groups	Treatment Description
I	Normal group	Was given a regular meal and 3 cc of distilled water daily for 16 weeks.
II	DMH	First 16 weeks, received DMH (20 mg/kg.b.w.) suspended in propylene glycol weekly s.c. (Ross et al., 1984).
III	DMH + Ph-NPs (25 mg/kg.b. w.)	Received DMH plus with Ph-NPs orally in a single daily dose for 16 weeks (Alamir et al., 2024), during the initial 16-week period.
IV	DMH + Ph-NPs (50 mg/kg.b. w.)	Received DMH plus Ph-NPs (50 mg/kg.b. w.) orally in a single daily dose for 16 weeks (Alamir et al., 2024), during the initial 16-week period.
V	DMH + 5-FU (20 mg/kg.b.w.)	Received 5-FU suspended in propylene glycol s.c. in a single weekly dose for 16 weeks (Boshra and Hussein, 2016a).
VI	DMH + 5-FU + Ph-NPs (50 mg/kg.b. w.)	The DMH plus 5-FU and Ph-NPs, in a single daily dose for 16 weeks.

### 2.4 Biochemical studies

On the 113th day the number of surviving mice were recorded for each groups. In the next day, blood samples (0.4 mL blood/mice) from the retro-orbital venous plexus of mice were collected in tubes containing heparin. We used one part for hematological examinations (Grindem, 2011) and centrifuged the second part at 1000 x*g* for 20 min. The obtained plasma was used for a cancer antigen 19–9 (CA 19–9) assay using an ELISA kit (ab99992, LDN Labor Diagnostika Nord, GmbH& Co., KG). We removed the colon tissues, rinsed them in an ice-cold 0.9 % sodium chloride solution, and divided them into two parts: We took the first part, washed it in distilled water, and homogenized it with a ten-fold amount of physiological saline using an electrical homogenizer at 3000 r.p.m. for 15 min at 4 °C. We stored the obtained supernatant at −20 °C until we estimated reduced glutathione (GSH), superoxide dismutase (SOD), and malondialdehyde (MDA) (Tsikas, 2017; Alghurab et al., 2024; Kakkar et al., 1984).

In addition, ELISA kits [*E*-EL-H0099, Elabscience Inc., Texas, USA; ab181421, Abcam, Texas, USA; MBS824788, MyBioSource, Inc., San Diego, CA; ab99992, Abcam, USA; EH3898, Wuhan Fine Biotech Co., Ltd., China] was used to estimate interleukin-2 (IL-2), tumor necrosis factor-alpha (TNF-α), transforming growth factor-beta (TGF-β1), carcinoembryonic antigen (CEA), Tumor protein (P53), respectively.

### 2.5 Molecular study

Under the recommendations provided by the manufacturer, we extracted total RNA from colon tissues weighing between 10 and 15 mg using Sepasol-RNA1Super. An Applied Biosystems 7500 Fast Real-Time PCR System (Foster City, CA, USA) was utilized to perform RT-PCR. The reaction volume was 25 μL, and 50 ng of RNA template was utilized for each reaction. The specific primers employed were 70 nM. Meridian Bioscience Inc./Bioline, which has its headquarters in Memphis, Tennessee, in the United States of America, is the manufacturer of the kit. The gene sequences are presented in Table 2.

**Table 2:** Primer sequences.

Gene	sequence of primers
PI3K	F:5′- AACACAGAAGACCAATACTC -3′ R:5′- TTCGCCATCTACCACTAC -3′
Akt	F:5′- GTGGCAAGATGTGTATGAG-3′ R:5′- CTGGCTGAGTAGGAGAAC-3′
β-actin (internal control for qRT-PCR)	F:5′- CACCCGCGAGTACAACCTT −3′ R:5′- CCCATACCCACCATCACACC -3

### 2.6 Histological examination

The second section of colon specimens was fixed in a 10 % formalin solution, we embedded the specimens in paraffin and dried them in graded alcohol. The tiny slices, which had a thickness of 5 μm, were stained with hematoxylin and eosin (H&E) and then placed on glass slides for light microscopic examination, employing the method proposed by Bancroft and Steven (Bancroft and Steven, 1983).

### 2.7 Statistical analysis

Using the SPSS/20 program, the data are presented as the mean plus or minus the standard deviation (Roa et al., 1985). To evaluate the hypotheses, we utilized the analysis of variance (ANOVA) test with a one-way design. An evaluation of statistical significance was carried out using *P* values that were lower than 0.05.

## 3 Results

### 3.1 Descriptive analysis of Ph-NPs

TEM micrographs of Ph-NPs are depicted in Figs. (1a and b). This was shown by the fact that their average vesicle size was 114.06 ± 8.35 nm with spherical, smooth, transparent nanoparticles in the shape.

However, Ph-NPs also had a polydispersity index (PDI) of 0.05, which means they had a narrow particle size distribution with a zeta potential value of −21.5 ± 0.8 mV. However, the percentage of phloretin entrapped within NSLs was 78.60 ± 0.32 % as tabulated in Table 3.

**Table 3:** Entrapment efficiency (%), particle size, Polydispersity index, and ZETA potential values of Ph- NPs.

Drug	Entrapment efficiency (%)	Particle size (nm)	Polydispersity index	ZETA potential (mv)
Ph-NPs	78.60 ± 0.32	114.06 ± 8.35	0.05	-21.5 ± 0.8

**Fig. 2:**
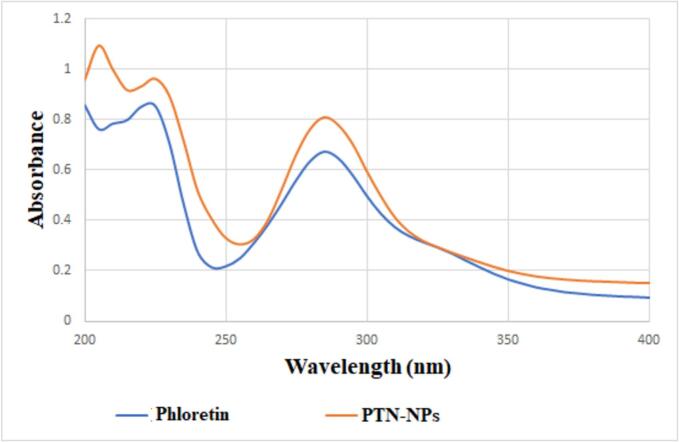
UV spectrum of Phloretin and PTN-NPs.

The absorption spectra of pure phloretin and Ph-NPs are depicted in Fig. 2. Band I (225 nm) corresponded to the benzoyl system, while band II (285 nm) represented the ring cinnamoyl system. Both bands were prominent in phloretin. Surface plasmon resonance (SPR) peaks at 225 and 285 nm are unique to Ph-NPs and can be seen in their absorption spectra.

**Fig. 3:**
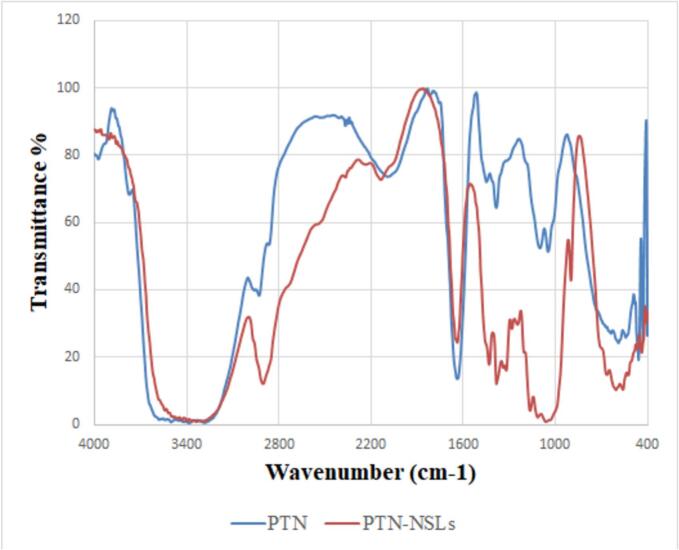
FT-IR spectrum of Phloretin and Ph-NPs.

Fig. 3 displays phloretin and Ph-NP FT-IR spectra. The presence of different functional groups caused peaks in the FT-IR spectra of phloretin. We identified these groups at 1382 cm-1 (O—H bending), 1639 cm^−1^ (C=O), 2927 cm^−1^ (CH-arom.), and 2861 cm^−1^ (CH-aliph.). Ph-NP spectra exhibited consistent characteristic peaks, albeit with slight variations in intensity.

### 3.2 Study of Ph-NP release

**Fig. 4:**
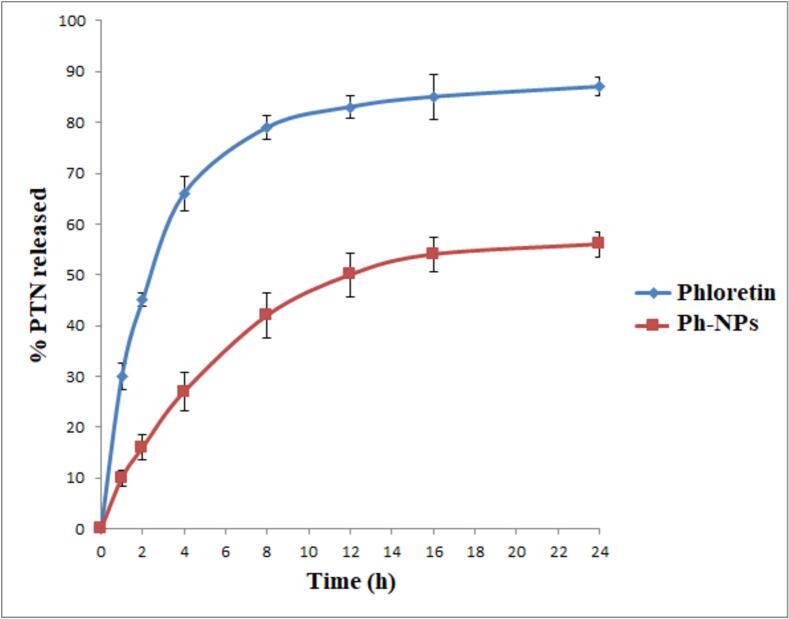
Drug release curve of Phloretin and Ph-NPs at PH 6.8.

Phloretin release from the Ph-NPs during 24 h was depicted in Fig. 4, with the temperature kept at (37 ± 0.5 °C) and pH at 6.8. The figure showed that there is a dissimilarity among free PTN and PTN-NSLs in the initial releasing rate through the first hour of shaking time; the free PTN release was rapid and increased constantly over time, and the percentage of the cumulative release was about 87.83 ± 1.84 % after 24 h from the beginning. Whereas the curve of PTN-NSLs showed a sustained release pattern with a slow rate, and the % release was 56.87 ± 2.45 % at the end of the experiment.

### 3.3 The viability and inhibitory values of Ph-NSLs against Caco-2 cell lines

**Fig. 5:**
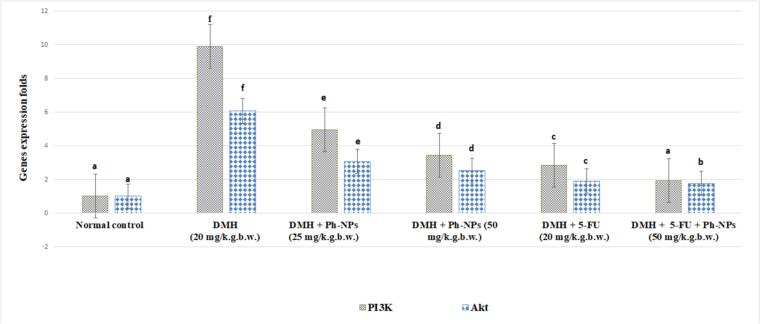
Effect of Ph-NPs, and 5-fluorouracil on colon PI3K and Akt gene expression levels in treated mice.

Table 4 and Fig. 5 explain the IC50, viability and inhibitory values of Ph-NSLs against Caco-2 cell lines. The IC50 values of Ph-NSLs were recorded at 15.76 ± 0.42 μg/ml. The cell viability and inhibitory values were recorded at 82.03 and 17.97 % at 0.8 μg. However, the cell viability and inhibitory values were recorded at 10.3 and 89.7 % at 50 μg. Moreover, the cell viability and inhibitory values were recorded at 2.39 and 97.61 % at 100 μg.

**Table 4:** Effect of Ph-NPs, and 5-fluorouracil on hemoglobin (Hb), red blood cells (RBCs), and white blood cell (WBCs) levels in treated mice.

No.	Groups	Hb (g/dl)	RBCs (×10^9^/uL)	WBCs (×10^6^/uL)
(I)	Normal control	14.28 ± 0.32^d^	4.98 ± 0.52^d^	6.42 ± 0.47^a^
(II)	DMH (20 mg/k.g.b.w.)	11.31 ± 0.91^b^	3.65 ± 0.45^b^	15.23 ± 1.06^f^
(III)	DMH + Ph-NPs (25 mg/k.g.b.w.)	12.37 ± 0.43 ^bc^	4.11 ± 0.40^bc^	11.36 ± 0.85^d^
(IV)	DMH + Ph-NPs (50 mg/k.g.b.w.)	13.63 ± 0.35^c^	4.67 ± 0.19^c^	8.19 ± 0.54^b^
(V)	DMH + 5-FU (20 mg/k.g.b.w.)	8.93 ± 0.39^a^	3.11 ± 0.22 ^a^	13.04 ± 0.50 ^e^
(VI)	DMH + Ph-NPs (50 mg) 5-FU (20 mg)	12.39 ± 0.32^bc^	4.26 ± 0.25^bc^	9.61 ±0.90 ^c^

Data shows the mean ± standard deviation of several observations within each treatment. Data followed by the same letter are not significantly different at P ≤ 0.05.

### 3.4 Effect of Ph-NSLs, and 5-fluorouracil on number of tumor/mice and average of tumor size in treated mice

Number of tumor/mice and average of tumor size in treated mice are illustrated in Table 6. When compared to DMH-injected mice (*p* < 0.05) number of tumor/mice and average of tumor size in Ph-NSLs (25 mg/kg b.w.)-treated mice were substantially decreased by 45.34, and 18.48 %, respectively. In addition, as compared to DMH-treated mice, administration of Ph-NSLs (50 mg/kg b.w.) considerably decreased number of tumor/mice and average of tumor size levels by 56.69, and 28.89 %, respectively. However, a group of mice treated with 5-FU (20 mg/kg b.w.) significantly reduced their number of tumor/mice and average of tumor size values by 69.84, and 58.61 %, respectively, compared to DMH-injected mice. Moreover, giving 5-FU and Ph-NSLs (50 mg/kg b.w.) together decreased the levels of number of tumor/mice and average of tumor size by 85.46, and 78.35 %, respectively, compared to mice that had been injected with DMH.

### 3.5 Effect of Ph-NPs and 5-FU on Hb, RBCs, and WBC levels in treated mice

In treated mice, plasma blood Hb, RBCs, and WBCs are explained in Table 4. When compared to normal mice, DMH-injected mice had a considerable drop (*p* < 0.05) in Hb and RBC levels by 20.29 and 26.70 %, respectively, as well as a significant rise in WBCs of 137.22 %. Furthermore, when compared to DMH-injected mice (p < 0.05), Hb and RBC levels in Ph-NPs (25 mg/kg b.w.)-treated mice were substantially increased by 9.37 and 12.60 %, respectively, as well as a significant decrease in WBCs by 245.41 %. In addition, as compared to DMH-treated mice, administration of Ph-NPs (50 mg/kg b.w.) considerably increased Hb and RBC levels by 20.51 and 27.95 %, respectively, while significantly decreasing WBCs by 46.22 % (*p* < 0.05). Conversely, a group of mice treated with 5-FU (20 mg/kg b.w.) significantly reduced their blood levels of Hb, RBCs, and WBCs compared to DMH-injected mice. Also, giving 5-FU and Ph-NPs (50 mg/kg b.w.) together raised the levels of Hb and RBCs in the blood by 9.54 and 16.71 %, respectively, while lowering the levels of WBCs by 36.90 % (p < 0.05) compared to mice that had been injected with DMH.

### 3.6 Effect of Ph-NSLs, and 5-fluorouracil on number of surviving mice

Number of surviving mice in treated mice are illustrated in Table 5. When compared to DMH-injected mice (*p* < 0.05) number of mice in Ph-NSLs (25 mg/kg b.w.)-treated mice were substantially increased by 50 %, respectively. In addition, as compared to DMH-treated mice, administration of Ph-NSLs (50 mg/kg b.w.) considerably increased number of of surviving mice levels by 66.67 %, respectively. However, a group of mice treated with 5-FU (20 mg/kg b.w.) significantly increased their number of of surviving mice values by 16.67 %, respectively, compared to DMH-injected mice. Moreover, giving 5-FU and Ph-NSLs,(50 mg/kg b.w.) together increased the levels of number of surviving mice by 20 %, respectively, compared to mice that had been injected with DMH.

**Table 5:** Effect of Ph-NPs, and 5-fluorouracil on colon GSH, SOD, and MDA levels in treated mice.

No.	Groups	GSH (mg/ 100 g tissue)	SOD (U/g tissue)	MDA (nmol/g tissue)
(I)	Normal control	68.42 ± 2.50^f^	302.75 ± 17.88^f^	8.36 ± 0.45^a^
(II)	DMH (20 mg/k.g.b.w.)	19.01 ±1.18 ^b^	136.59 ±8.01^b^	73.48 ±6.08^f^
(III)	DMH + Ph-NPs (25 mg/k.g.b.w.)	37.58 ± 3.56^c^	179.00 ± 11.77^c^	21.55 ± 2.03 ^c^
(IV)	DMH + Ph-NPs (50 mg/k.g.b.w.)	54.96 ± 3.54^e^	263.46 ± 17.64^e^	15.38 ± 1.47^b^
(V)	DMH + 5-FU (20 mg/k.g.b.w.)	15.35 ± 1.00^a^	95.54 ± 6.96^a^	58.93 ± 3.89 ^e^
(VI)	DMH + Ph-NPs (50 mg) 5-FU (20 mg)	42.65 ± 2.88^d^	238.10 ± 18.45^d^	41.88 ± 4.53^d^

Data shows the mean ± standard deviation of several observations within each treatment. Data followed by the same letter are not significantly different at P ≤ 0.05.

### 3.7 Effect of Ph-NPs and 5-FU on GSH, SOD, and MDA levels in the colon of treated mice

Colon GSH and SOD levels significantly decreased in DMH-injected mice by 72.21 and 54.88 %, respectively, while MDA levels significantly increased by 778.94 % Table 5. The colon GSH and SOD levels were significantly higher (*p* < 0.05) in mice treated with Ph-NPs (25 mg/kg b.w.) compared to mice injected with DMH. The MDA level was also significantly lower, at 70.76 %. In mice given Ph-NPs (50 mg/kg b.w.), colon GSH and SOD levels went up significantly (p < 0.05) by 189.11 and 92.88 %, respectively. MDA levels went down significantly by 179.06 % compared to DMH-injected mice, MDA levels decreased significantly by 179.06 %. However, giving 5-FU (20 mg/kg b.w.) significantly decreased colon GSH, SOD, and MDA levels by 19.25, 30.05, and 19.80 %, respectively. Compared to the DMH-injected mice, the 5-FU and Ph-NPs (50 mg/kg b.w.) treatments also increased GSH and SOD levels in the colon by 124.35 and 74.32 %. However, it also significantly decreased MDA levels by 43.00 % (p < 0.05).

### 3.8 Effect of Ph-NPs and 5-FU on IL-2, TNF-α, and TGF-β1 levels in the colon of treated mice

When compared to normal mice, mice that were injected with DMH had 144.71 times higher levels of colon IL-2, 343.09 times higher levels of TNF-α, and 158.58 times higher levels of TGF-β1, Table 6. Also, giving Ph-NPs (25 mg/kg b.w.) to mice that had been injected with DMH significantly decreased the levels of IL-2, TNF-α, and TGF-β1 in their colons by 23.30, 25.01, and 31.71 %, respectively (*p* < 0.05). When mice were given Ph-NPs (50 mg/kg b.w.), the levels of colon IL-2, TNF-α, and TGF-β1 were compared to mice that were injected with DMH, they were lowered by 45.25, 53.84, and 48.45 %, respectively.

**Table 6:** Effect of Ph-NPs, and 5-fluorouracil on colon IL-2, TNF- α, and TGF-β1 levels in treated mice.

No.	Groups	IL-2 (pg/ g tissue)	TNF- α (Pg/ g tissue)	TGF-β1 (pg/ g tissue)
(I)	Normal control	5.68 ± 0.44 ^a^	34.69 ± 3.22^a^	34.07 ± 3.10^a^
(II)	DMH (20 mg/k.g.b.w.)	13.90 ±1.06 ^e^	153.71 ±8.80^f^	88.10 ±4.31^f^
(III)	DMH + Ph-NPs (25 mg/k.g.b.w.)	10.66 ± 0.91^d^	115.27 ± 12.60^e^	60.16 ± 5.54 ^e^
(IV)	DMH + Ph-NPs (50 mg/k.g.b.w.)	7.62 ± 0.23^b^	70.95 ± 5.33 ^c^	44.53 ± 3.68 ^c^
(V)	DMH + 5-FU (20 mg/k.g.b.w.)	9.22 ± 0.45^c^	84.67 ± 9.21^d^	51.07 ± 5.99^d^
(VI)	DMH + Ph-NPs (50 mg) 5-FU (20 mg)	6.51 ±0.30^a^	44.81 ± 4.54 ^b^	37.96 ±3.70^b^

Data shows the mean ± standard deviation of several observations within each treatment. Data followed by the same letter are not significantly different at P ≤ 0.05.

When 5-FU (20 mg/kg b.w.) was given to mice instead of DMH-injected mice, levels of colon IL-2, TNF-α, and TGF-β1 were 33.66, 44.92, and 42.03 % lower, respectively. Colon IL-2, TNF-α, and TGF-β1 levels dropped by 52.13, 70.84, and 56.91 % in mice that had been injected with DMH and then given 5-FU and Ph-NPs (50 mg/kg b.w.) together.

### 3.9 Effect of Ph-NPs, and 5-FU on colon P53, CEA, and plasma C19.9 levels in colon of treated mice

Lower levels of P53 (*p* < 0.05) were found by 62.0 %, while levels of CEA and C19.9 were found to be 460.62 and 299.16 % higher in DMH-injected mice compared to normal mice, Table 7. Also, the levels of P53 went up by 77.32 % and those of CEA and C19.9 went down by 30.64 and 30.43 %, respectively, in mice that were given Ph-NPs (25 mg/kg b.w.) compared to mice that were given DMH (Table 7) (*p* < 0.05). Giving Ph-NPs (50 mg/kg b.w.) to mice that have been injected with DMH increases P53 levels by 135.12 % and lowers CEA and C19.9 % levels by 48.8 % and 48.35 %, respectively, compared to mice that have been injected with DMH (p < 0.05). Compared to mice that were injected with DMH, mice that were given 5-FU (20 mg/kg/b/w) had a significant rise in P53 levels by 101.95 % and a significant drop in CEA and C19.9 levels by 60.53 and 40.90 %, respectively. This study also found that giving 5-FU and Ph-NPs (50 mg/kg b.w.) together increased P53 levels by 150 % and decreased CEA and C19.9 levels by 74.32 and 63.64 % compared to mice that had been injected with DMH (p < 0.05).

**Table 7:** Effect of Ph-NPs, and 5-fluorouracil on colon P53, CEA, and plasma C19.9 levels in treated mice.

No.	Groups	P53 (ng/ g tissue)	CEA (ng/g tissue)	C19.9 (U/mL)
(I)	Normal control	10.97 ± 1.19 ^d^	4.85 ± 0.35^a^	15.05 ± 0.96 ^a^
(II)	DMH (20 mg/k.g.b.w.)	4.10 ± 0.30 ^a^	27.19 ±2.10 ^f^	61.87 ±4.35 ^f^
(III)	DMH + Ph-NPs (25 mg/k.g.b.w.)	7.27 ± 0.44^b^	18.86 ± 1.19^e^	43.04 ± 2.20 ^e^
(IV)	DMH + Ph-NPs (50 mg/k.g.b.w.)	9.64 ± 0.52^c^	13.92 ± 0.70 ^d^	31.95 ± 2.96 ^c^
(V)	DMH + 5-FU (20 mg/k.g.b.w.)	8.28 ± 0.45 ^b^	10.73 ± 0.80 ^c^	36.56 ± 3.21 ^d^
(VI)	DMH + Ph-NPs (50 mg) 5-FU (20 mg)	10.25 ±0.34^d^	6.98 ± 0.56 ^b^	22.49 ± 2.41^b^

Data shows the mean ± standard deviation of several observations within each treatment. Data followed by the same letter are not significantly different at P ≤ 0.05.

### 3.10 Effect of Ph-NPs, and 5-fluorouracil on colon PI3K and Akt gene expression levels in treated mice

Compared to normal mice, DMH-injected mice have substantially increased (*p* < 0.05) colon PI3K and Akt gene expression levels by 880.19 and 500.98 %, respectively. When mice were given Ph-NPs (25 mg/kg b.w.), their PI3K and Akt gene expression levels dropped significantly compared to mice that were injected with DMH (*p* < 0.05). When DMH injected mice are given Ph-NPs (50 mg/kg b.w.), the levels of the PI3K and Akt genes in their colon drop by 65.05 and 58.22 %, respectively. Compared to mice that were injected with DMH, mice that were given 5-FU (20 mg/kg/b/w) had a significant drop in PI3K and Akt gene expression levels by 71.41 and 68.58 %, respectively. In addition, administration of 5-FU and Ph-NPs (50 mg/kg b.w.) together decreased PI3K and Akt gene expression levels by 80.60 and 70.88 %, respectively (p < 0.05), Fig. 5.

### 3.11 The influence of Ph-NPs and 5-FU on the histological changes that occurred in the colon of treated mice

**Fig. 6:**
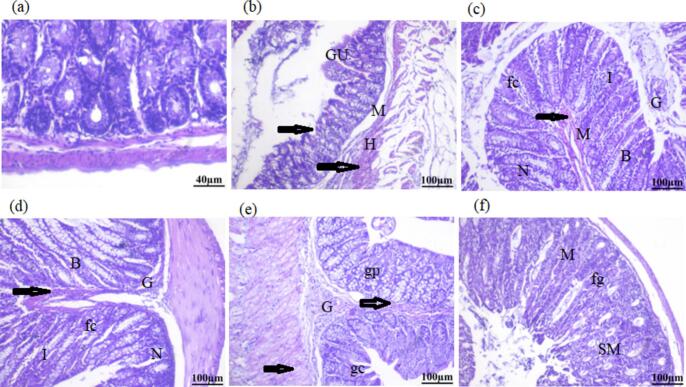
Histological examination of colon sections. **(a)**: Group I, normal group; **(b)**: Group II: Was injected with DMH (20 mg/k.g.b.w.); (**c)**: Group III: Was injected with DMH (20 mg/k.g.b.w.) and administrate with Ph-NPs (25 mg/kg.b.w.); **(d):** Group IV: Was injected with DMH (20 mg/k.g.b.w.) and administrate with Ph-NPs (50 mg/kg.b.w.); **(e)**: Was injected with DMH (20 mg/k.g.b.w.) and administrate with 5-FU (20 mg/kg.b.w.); **(f):** Was injected with DMH (20 mg/kg.b.w.) and administrate with Ph-NPs (50 mg/kg.b.w.) + 5-FU (20 mg/kg.b.w.)

Under a microscope, sections stained with H&E showed a normal arrangement of the colon parenchyma (Fig. 6a). A normal control group-I appearance was observed in the tissue. Colon sections from group II, the group that received DMH, showed atrophic, disordered fundic glands (gastric ulcer) and pyknotic nuclei (G). The colon sections also showed extremely poorly broken-up colon mononuclear cell infiltrations (arrows). Goblet cells have a smaller number (shown by the arrowhead). We investigated a hemorrhagic area (H). (Fig. 6 c & d) Lymphatic cells infiltrated the muscle layer (M) arrangement, as indicated by the arrow, causing greater damage to this particular group than to the other control groups. The colon sections obtained from the Ph-NPs at doses of 25 and 50 mg/kg body weight showed a consistent distribution of the fundic glands. We observed that the mucosal layer, submucosa, and muscularis externa were all in good condition. Additionally, we found the isthmus (I), neck (N), and base (B) to be in good condition. Fig. 6, shows a colon segment from mice in group V. The mice in Group V received a dose of 5FU at 20 mg/kg body weight. Goblet cells (GC), gastric pit cells (GP), and mononuclear cells (arrow) were visible in the region between the bottom sections of the stomach glands. This included the presence of fundic glands (fg), submucosa (SM), and muscularis (M) (Fig. 6f).

## 4 Discussion

Colorectal cancer (CRC) is a significant public health concern, ranking third in terms of prevalence in both developed and developing nations among cancer-causing disorders (da Paz et al., 2012). Several studies proved that the application of synthetic and natural products in the treatment of different diseases (El Gizawy et al., 2021; Gobba et al., 2018; Borik and Hussein, 2021; Boshra and Hussein, 2016b). In addition, several documents reported the anticancer activity of nanoparticles from natural products (Mostafa et al., 2023; Soliman et al., 2022). In the present study, Ph-NPs were prepared to estimate their anticancer activity against colon cancer in mice.

The Ph-NPs obtained in our study are elastic nanovesicles that may be used as drug-delivery vehicles since they can accommodate a variety of drug compounds. Ph-NPs have a growing interest in a variety of administration routes (Judy et al., 2021). These are amphiphilic because a non-ionic surfactant forms a vesicle to contain the drug (Lalu et al., 2017). The extremely small and microscopic size of Ph-NPs improves their capacity to pass through biological membranes for oral delivery (Mohamad et al., 2022; Mazyed et al., 2021). Our results showed that the synthesized Ph-NPs were uniform and spherical, well dispersed without any aggregation observed, and had relatively smooth surfaces. Morphological examination confirmed the former vesicle size values obtained by DLS. The TEM results emphasized the best possible formulation. Ph-NPs are usually round and don't tend to stick together. This means that the particles have small diameters, which makes it easier for them to pass through biological membranes for oral delivery. The low value of PDI demonstrated that the particle size of Ph-NPs was distributed homogeneously. A high negative or positive zeta potential means that the nanovesicles are pushing against each other, which creates a steady colloidal dispersion and the formation of a suspension that doesn't clump together (Jagwani et al., 2020). A high negative zeta potential value was found for the improved Ph-NPs. This meant that the nanovesicles were physically stable and could be used to deliver drugs orally since the zeta potential drops as the drug load increases (Alamir et al., 2024). This might be because phloretin interacts with the head group of surfactants, which stops the negative charges on the noisome surface from being negative. This observation was also reported previously: drugs could reduce the negative surface charges (El Gizawy et al., 2021).

UV–vis PTN's spectra are typical for flavones, and strong absorption maxima are seen at wavelengths between 200 and 400 nm. Both the ring benzoyl system (typically 200–270 nm) and the ring cinnamoyl system (300–380 nm) are frequently referred to as these bands (Dhamecha et al., 2016). Our results showed two prominent bands at 225 and 285 nm in the case of phloretin. On the other hand, the UV–Vis absorption spectra of Ph-NPs exhibited two bands at 225 and 285 nm. The UV–Vis spectra did not show any significant changes in the absorption spectra of Ph-NPs compared to pure phloretin. The outcomes were identical to those that Mohamad et al. (Mohamad et al., 2022) reported. They said that the UV–vis spectra of gallic acid-niosomes did not show any major differences in their absorption spectra when compared to free gallic acid. They have a strong absorption at 280 nm.

We checked how much phloretin was stuck inside the nanoparticles to show that it has strong hydrogen-bonding groups (-OH, -C=O), enough chain adaptability, and the ability of the customized NSL nanoplatforms to hold a lot of phloretin. This is important because it could be used to improve oral delivery (Saeting et al., 2022). FTIR analysis confirmed that the successful compatibility of Ph-NPs confirms the chemical stability of the formula. When we looked at the FTIR spectra of AKBA and AKBA-loaded spanlastic nanovesicles (SNVs), we saw that the two mixtures worked well together, which backed up what Badria and Mazyed (Badria and Mazyed, 2020) reported. At pH 6.8, free phloretin and Ph-NPs revealed a drug release of around 87.83 ± 1.84 % and 56.87 ± 2.45 %, respectively, within 24 h. The idea that free phloretin has poor efficiency and low cellular bioavailability, which restricts its use for oral delivery, supported these findings (Sezgin-Bayindir and Yuksel, 2012). So, the improved Ph-NP formulation should make oral delivery more effective, increase bioavailability, and improve penetration (Tsikas, 2017). In agreement with Alamir et al. (Alamir et al., 2024), who stated that the release of phenol-nanostructured lipid carriers had a significant sustained effect compared with a phloretin ethanolic solution.

In the current investigation, Ph-NSLs have shown promising cytotoxic activity against Caco-2 cells with low value IC50. These results are important as they help in understanding the potential of Ph-NSLs as a therapeutic agent for cancer treatment.

Our results were confirmed with reported by Trifan, and Luca, (Thrall et al., 2012), who showed that phloretin nanoparticles exhibited significant cytotoxic effects on Caco-2 cells. The nanoparticles were able to induce cell death through mechanisms such as apoptosis, which is a programmed cell death process crucial for eliminating cancer cells. The study highlighted that Ph-NSLs could effectively inhibit cell proliferation and induce apoptosis by modulating various signaling pathways.

The study investigated the effects of Ph-NSLs and 5-FU on the survival of mice with DMH-induced colon cancer. The results showed that treatment with Ph-NSLs significantly increased the number of surviving mice in a dose-dependent manner, with higher doses being more effective. In contrast, 5-FU also improved survival but to a lesser extent. Interestingly, the combination of 5-FU and Ph-NSLs resulted in a moderate increase in survival, suggesting potential interactions between the treatments. These findings highlight the potential of Ph-NSLs as a therapeutic agent and the need for further optimization of combination therapies.

Our results proved the significant reduction in tumor size and number in mice tread with Ph-NSLs and 5-FU, indvudally.

The dose-dependent increase in survival suggests that Ph-NSLs are effective in reducing tumor burden and improving overall health outcomes. While 5-FU is a well-established chemotherapeutic agent, its effectiveness in increasing survival rates is comparatively lower than that of Ph-NSLs. This highlights the potential of Ph-NSLs as a more potent alternative or complementary treatment to traditional chemotherapy. The combination of 5-FU and Ph-NSLs, although showing a moderate increase in survival, indicates the possibility of optimizing combination therapies. This approach could leverage the strengths of both treatments, potentially leading to better therapeutic outcomes.

These points underscore the promising role of Ph-NSLs in enhancing survival in colon cancer models and the need for further research to optimize treatment strategies.

Our results were in align with findings from other study on phloretin's anticancer properties that highlighted that phloretin significantly reduces tumor growth by modulating various signaling pathways, including apoptosis and cell proliferation (Hişmioǧulları et al., 2013). Our study also noted that elevation of Ph-NSLs's effectiveness against cancer when used in combination with other chemotherapeutic agents, enhances their efficacy by reversing drug resistance and improving therapeutic outcomes. This synergistic effect is similar to the observed results in your study, where the combination of 5-FU and Ph-NSLs led to a more substantial reduction in tumor size and number compared to either treatment alone.

To assess the anticancer activity of Ph-NPs in the ongoing study, we employed mice that had received DMH treatment. According to one report, DMH injections for sixteen weeks caused colon cancer in mice as a model for the disease (Ross et al., 1984).

In the present study, the injection of mice with DMH depleted the Hb and RBCs as well as elevated WBCs levels dramatically. Our findings were like those of Ross et al. (Ross et al., 1984). They found that giving DMH to mice causes' inflammation, lowers the levels of Hb and RBCs, raises WBCs, and causes colon cancer.

Ph-NPs were revealed to have unique anti-inflammatory properties (Alamir et al., 2024), as they significantly reduced the inflammation and elevated levels of Hb and RBCs as well as depleted WBCs levels in DMH-treated mice.

The decreased Hb and RBCs levels observed in the present study were probably due to the harmful effects of DMH on bone marrow and hematopoietic organs. Anemia was seen in this study, and a lower RBC count may have been linked to a slower rate of erythrogenesis. This is because DMH hurts erythropoiesis, which means that erythrocytes have a shorter life span and their membranes break down, which turns them into echinocytes (Thrall et al., 2012). On the other hand, Ross et al. (Ross et al., 1984) found that the administration of natural products altered the hematological parameters of experimental rats. The current study observed a significantly lower white blood cell count in the Ph-NPs group compared to the DMH group.

Given the anti-inflammatory properties of Ph-NPs (Alamir et al., 2024), we expected the observed decrease in WBC. Hişmioŧulları et al. (Hişmioǧulları et al., 2013) also reported that the WBC count was decreased in rats given flavonoids. This study's most significant finding was the improvement of some DMH-induced hematological changes in the group that received Ph-NPs with DMH. These findings show that Ph-NP treatment may help protect bone marrow and hematopoietic progenitor cells from changes caused by DMH.

On the other hand, the DMH-administered group showed considerably high levels of colon MDA. Also, the levels of GSH and SOD showed significantly low levels. In DMH-injected mice, ROS is produced, which induces colon oxidative damage (Alamir et al., 2024; Hişmioǧulları et al., 2013). Ph-NPs were shown to reduce ROS production and normalize the MDA, GSH, and SOD levels when administered to DMH-treated mice. Supplementing Ph-NPs helped the colon cells maintain antioxidant enzyme balance in DMH-treated mice. However, the administration of 5FU + DMH significant decrease in the colon GSH, and SOD levels as well as a significant increase in colon MDA as compared to double agents therapy Ph-NPs + 5-FU possibly due to the oxidative stress of 5-FU (Boshra and Hussein, 2016a).

A lot of studies in living things and in the lab have shown that phenolic compounds boost the effects of antioxidants like GSH, SOD, GPx, and GR in a wide range of cancers and diseases by lowering lipid peroxidation (Cheng et al., 2021; Nakajima et al., 2002). The results we got agreed with those of Beites et al. (Beites et al., 2021), who showed that injecting phenolics lowered MDA levels and increased the activity of antioxidant enzymes compared to the control group. Free radicals, such as superoxide or H_2_O_2_, can cause lipid peroxidation, which increases enzyme inactivation.

Our results provide evidence in favor of the theory that mice treated with DMH produced more inflammatory mediators due to the considerable increase in lipid peroxidation. We found that Ph-NPs may effectively lower inflammation by removing free radicals, lowering lipid peroxidation, increasing the production of antioxidant enzymes, and restoring normal levels of IL-2, TNF-α, and TGFβ1.

TGFβ1 controls the activities of inflammatory genes like IL-2 and TNF-α, which is a specific way that it links inflammation to cancer. Many toxic compounds have a direct toxic effect on the epithelium, destroy the mucosal barrier, and cause the elevated secretion of pro-inflammatory cytokines such as IL-2 and TNF-α (Al-Thakafy et al., 2024; Roa et al., 1985). The higher levels of IL-2 and TNF-α found by immunohistochemistry match the higher levels found in animals that were given DMH. The elevation of inflammatory mediators during DMH-induced oxidative stress is strongly associated with colon cancer pathogenesis. The current findings support those of El Gizawy et al. (El Gizawy et al., 2021), who claimed that the anti-inflammatory effects of several phenolics decreased the levels of cytokines. The administration of PTN-NSLs affected ROS, glutathione metabolism, IL-2, TNF-α, and TGFβ1. On the other hand, administration of Ph-NPs and 5-FU in combination resulted in a significant decrease in colon IL-2, TNF-α, and TGFβ1 levels as compared to single-agent therapy with 5-FU, possibly due to the anti-inflammatory properties of Ph-NPs and their combined effects with 5-FU (Alamir et al., 2024).

There is a significant relationship between cancer cells' adhesion to the lining of blood vessels and the presence of tumor markers such as P53, CEA, and CA19–9. Initially isolated as an antigen associated with colon cancer, it now serves as a tumor marker in gastrointestinal infections (Dong et al., 2013). Elevated serum CA19–9 levels are an adverse prognostic factor in colon cancer. This is because elevated serum CA19–9 levels are associated with a higher frequency of metastases and shorter survival rates (Hassan et al., 2023).

In the present study, mice treated with DMH reduced colon P53 levels as well as elevated CEA and C-19-9 levels in colon tissue. It was the same as what Sisik et al. (Dong et al., 2013) and Hassan et al. (De-Souza and Costa-Casagrande, 2018) found when they treated mice with DMH. They found that the treatment increased CEA levels and decreased P53 gene expression.

The current study found that giving Ph-NPs and 5-FU together led to a significant rise in colon P53 levels and a decrease in colon CEA and serum CA19–9 levels compared to giving 5-FU alone. This might be because Ph-NPs have antiproliferative properties that work better with 5-FU.

Furthermore, DMH administration causes tumors to develop in treated mice's colons. De-Souza and Costa-Casagrande (De-Souza and Costa-Casagrande, 2018), who reported the carcinogenic properties of DMH in the colon tissue of mice, confirmed our results. Understanding the mechanism of DMH primarily involves altering the DNA of stem colonocytes at the base of the intestinal crypts, a process that triggers the growth of colon adenocarcinomas (Umesalma and Sudhandiran, 2010). When DMH enters cells, it increases PI3K/Akt and Wnt, two oncogenic pathways that lead to the growth of colonic neoplasms (Yu et al., 2017). Akt is a key player in the growth of colon tumors; it stops GSK3β/APC/axin from breaking down β-catenin and increases the expression of oncogenes like c-Myc and cyclin D1 that target β-catenin (Shojaei-Zarghani et al., 2020).

The expression levels of the PI3K and Akt genes in the colon went downregulated significantly when Ph-NPs and 5-FU were given together. This effect was more pronounced than that observed with 5-FU administered as a single agent. This may be attributed to the antiproliferative characteristics of Ph-NPs and their synergistic effects with 5-FU. Notably, Ph-NPs have been found to affect the expression of several genes including cytokine-coding genes, and growth factors (Alamir et al., 2024).

Moreover, Ph-NPs can modulate the activity of PI3K/AKT signaling pathway as shown in the present study. As mentioned in several studies, the regulatory effects of phenolic compounds on the activity of the PI3K/AKT pathway have been better appraised in different contexts. When there is a neoplastic condition, Ph-NPs not only stop cells from behaving in a cancerous way and speed up the epithelial-mesenchymal transition, but they also make cancer cells more sensitive to drugs like 5FU. Therefore, we preferred the administration of Ph-NPs and 5 FU in combination to enhance the efficacy of their anti-cancer activity. The effects of Ph-NPs in suppressing the growth of cancer have been validated in some types of cell lines, such as prostate, lung, oral, breast, and liver (Nakhate et al., 2022).

A lot of research has shown that giving Ph-NPs to mice that have been injected with DMH along with 5FU makes the antiproliferative effects of Ph-NPs even stronger (Fayed et al., 2024). Stimulation also makes antioxidant enzymes work better and restores inflammatory mediators that have been depleted (El-Gizawy and Hussein, 2015). This is more proof that stimulation has a strong anti-cancer effect on Ph-NPs (Mosaad et al., 2022; Aziz et al., 2024; Al-Thakafy et al., 2024; Al-Mhanaa et al., 2024).

We examined the colon tissue of all the test animals using histopathology to see if colon carcinoma developed in the control group of carcinogens and to assess the effectiveness of Ph-NPs as a chemotherapy drug against DMH-induced colon cancer. The carcinogen control group developed hyperplastic lesions on the mucosal layer of colon tissue. In addition, the carcinogen control group had a larger area of bleeding in the hyperplastic lesion with full mucosal dysplasia. The treatment with Ph-NPs makes the area that is bleeding a lot smaller and returns the cells in the colon tissue to their normal structure. After being treated with Ph-NPs, the hyperplastic lesion got a lot smaller, and the goblet cells went back to their normal shape. These incidences show that Ph-NPs have anticancer activity against DMH-induced colon cancer in mice.

## 5 Conclusions

The TEM micrographs revealed that Ph-NSLs are spherical, smooth, and transparent nanoparticles. They exhibited a narrow size distribution and a stable zeta potential. Phloretin was efficiently entrapped within the NSLs. Absorption spectra showed distinct SPR peaks, while FT-IR spectra confirmed the presence of characteristic functional groups. Phloretin release studies indicated a sustained release from Ph-NSLs over 24 h, compared to the rapid release of free phloretin.

However, Ph-NSLs show strong cytotoxic effects against Caco-2 cell lines, significantly reducing cell viability and demonstrating high inhibitory potential. This highlights their promise as an effective anticancer agent. Moreover, The combination of 5-FU and Ph-NSLs resulted in a moderate increase in survival and significantly reduces tumor size and number, showing enhanced anticancer efficacy compared to individual treatments. This synergistic approach offers a promising strategy for improved cancer therapy.

The current study involved the preparation of Ph-NSLs to specifically target colon cancer in mice treated with DMH. The current study demonstrated that Ph-NSLs exert their anticancer effects by altering the activity of the pathway mediated by PI3K and AKT, activating P53, inhibiting CEA and C 19–9, and enhancing antioxidant and inflammatory indicators. In addition, the combination of Ph-NSLs and 5-FU resulted in a significant increase in colon P53 levels, along with a decrease in serum CA19–9 and colon CEA levels. Furthermore, the expression levels of PI3K/AKT genes were also reduced compared to treatment with 5-FU alone. The inhibitory actions of Ph-NSLs on cell growth and their synergistic effects with 5-FU may explain this phenomenon. The results of our study indicate that Ph-NSLs have the potential to be an effective and promising target for the treatment of colon cancer in mice.

## Ethics statements

The Faculty of Applied Health Sciences Technology at October 6 University, which received approval with the number 20210305, strictly adhered to the recommendations made by the Ethics Committee for the purpose of conducting research on animals and providing care for them.

## CRediT authorship contribution statement

**Ebtsam A. Abdel-Wahab:** Writing – original draft, Resources, Formal analysis, Data curation. **Zahraa Haleem Al-Qaim:** Investigation, Funding acquisition, Formal analysis. **Ahmed T.H. Faris Al-Karkhi:** Conceptualization, Data curation, Investigation, Methodology, Validation, Writing – review & editing. **Aysam M. Fayed:** Writing – review & editing, Writing – original draft, Visualization, Validation, Supervision, Software, Resources, Project administration, Investigation, Formal analysis, Data curation, Conceptualization. **Ahmed M. Eldmrdash:** Validation, Resources, Data curation, Conceptualization. **Mohammed Abdalla Hussein:** Software, Resources, Project administration, Methodology. **Amal Abdel-Aziz:** Writing – original draft, Supervision, Resources, Investigation. **Azza M. Metwaly:** Writing – review & editing, Validation, Methodology, Data curation, Conceptualization. **Heba.G. Abdelzaher:** Writing – review & editing, Writing – original draft, Visualization, Validation, Supervision, Resources, Investigation, Data curation, Conceptualization. **M.A. Abdelzaher:** Writing – review & editing, Writing – original draft, Validation, Supervision, Resources, Investigation, Funding acquisition, Formal analysis, Data curation, Conceptualization. **Diana A. ALsherif:** Visualization, Software, Resources, Investigation, Data curation, Conceptualization.

## Declaration of competing interest

The authors declare that they have no known competing financial interests or personal relationships that could have appeared to influence the work reported in this paper.

## Data Availability

Data will be made available on request.
